# Coexistence of plasmid-mediated quinolone resistance (PMQR) and extended-spectrum beta-lactamase (ESBL) genes among clinical *Pseudomonas aeruginosa* isolates in Egypt

**DOI:** 10.1186/s12866-024-03319-z

**Published:** 2024-05-21

**Authors:** Soha S. Abdelrahim, Noha A. Hassuna, Nancy G. F. M. Waly, Dalia N. Kotb, Haitham Abdelhamid, Shaimaa Zaki

**Affiliations:** 1 Department of Medical Microbiology and Immunology, Faculty of Medicine, Minia University, Minia, 61511 Egypt; 2 Department of Microbiology and Immunology, Faculty of Pharmacy, Minia University, Minia, Egypt; 3 Tropical Medicine Department, Faculty of Medicine, Minia University, Minia, Egypt

**Keywords:** *Pseudomonas aeruginosa*, Fluoroquinolones, PMQR, *Acc(6')-Ib-cr*, *Qnr*, ESBL, Egypt

## Abstract

**Background:**

Data about the prevalence of plasmid-mediated quinolone resistance (PMQR) and extended-spectrum beta-lactamase (ESBL) production in *P*. *aeruginosa* compared to the *Enterobacteriaceae* family is limited. The availability of limited therapeutic options raises alarming concerns about the treatment of multidrug-resistant *P*. *aeruginosa*. This study aimed to assess the presence of PMQR and ESBL genes among *P*. *aeruginosa* strains.

**Methods:**

Fifty-six *P. aeruginosa* strains were isolated from 330 patients with different clinical infections. Phenotypically fluoroquinolone-resistant isolates were tested by PCR for the presence of six PMQR genes. Then, *blaTEM, blaSHV*, and *blaCTX-M* type ESBL genes were screened to study the co-existence of different resistance determinants.

**Results:**

Overall, 22/56 (39.3%) of the studied *P. aeruginosa* isolates were phenotypically resistant to fluoroquinolones. PMQR-producing *P. aeruginosa* isolates were identified in 20 isolates (90.9%). The *acc(6')-Ib-cr* was the most prevalent PMQR gene (77.3%). The *qnr* genes occurred in 72.7%, with the predominance of the *qnrA* gene at 54.5%, followed by the *qnrS* gene at 27.3%, then *qnrB* and *qnrC* at 22.7%. The *qepA* was not detected in any isolate. The *acc(6')-Ib-cr* was associated with *qnr* genes in 65% of positive PMQR isolates. Significant differences between the fluoroquinolone-resistant and fluoroquinolone-susceptible isolates in terms of the antibiotic resistance rates of amikacin, imipenem, and cefepime (*P* value < 0.0001) were found. The ESBL genes were detected in 52% of cephalosporin-resistant *P. aeruginosa* isolates. The most frequent ESBL gene was *blaCTX-M* (76.9%), followed by *blaTEM* (46.2%). No isolates carried the *blaSHV* gene. The *acc(6')-Ib-cr* gene showed the highest association with ESBL genes, followed by the *qnrA* gene. The correlation matrix of the detected PMQR and ESBL genes indicated overall positive correlations. The strongest and most highly significant correlation was between *qnrA* and *acc(6')-Ib-cr* (*r* = 0.602) and between *qnrA* and *blaCTX-M* (*r* = 0.519).

**Conclusion:**

A high prevalence of PMQR genes among the phenotypic fluoroquinolone-resistant *P. aeruginosa* isolates was detected, with the co-carriage of different PMQR genes. The most frequent PMQR was the *acc(6')-Ib-cr* gene. Co-existence between PMQR and ESBL genes was found, with 75% of PMQR-positive isolates carrying at least one ESBL gene. A high and significant correlation between the ESBL and PMQR genes was detected.

**Supplementary Information:**

The online version contains supplementary material available at 10.1186/s12866-024-03319-z.

## Introduction

*Pseudomonas aeruginosa (P. aeruginosa*) is an opportunistic gram-negative bacterium that can inhabit moist environments in healthcare settings and colonize about 50% of hospitalized patients [1]. Strains of *P. aeruginosa* can survive on minimal nutritional requirements and adapt to a variety of physical conditions, allowing this organism to persist in both hospital settings and the community [2]. *P. aeruginosa* is one of the top-listed bacteria causing 10–11% of nosocomial infections, such as urinary tract infections (UTI), surgical site infections, burn wound infections, pneumonia, and bloodstream infections [1]. Recently, *P. aeruginosa* has been considered one of the most challenging pathogens for drug resistance. The World Health Organisation (WHO) classified *P. aeruginosa* on the critical priority list of pathogens that need urgent development of new antibiotics to treat their infections [3].

Heavy use of broad-spectrum antibiotics in the last decades led to the emergence of multiple drug-resistant (MDR) strains of *P. aeruginosa*, resulting in an increased length of hospital stay, increased healthcare costs, and high morbidity and mortality rates. A high prevalence of MDR *P. aeruginosa* was reported in Egypt and neighboring countries due to improper use of antimicrobial agents because of the negative population’s attitudes and lack of policies for antibiotic prescription [4].

Fluoroquinolones are potent synthetic antimicrobial agents that are active against a wide range of gram-negative and gram-positive bacteria. They are classified, according to their discovery history and their antibacterial properties, into four generations. Second- and third-generation drugs (ciprofloxacin and levofloxacin) are commonly used in clinical practice. The rapid global rise in the prevalence of fluoroquinolone-resistant strains has become a major concern in the past few years due to the heavy use of quinolones in human, veterinary, and agricultural medicine [5].

There are two mechanisms mediating fluoroquinolone resistance. The first is chromosomal mutations in genes encoding DNA gyrase and topoisomerase IV enzymes that are the quinolone targets. Before 30 years, the only known resistance mechanisms to quinolone resistance were chromosomally mediated. The second mechanism is plasmid-mediated quinolone resistance (PMQR), which appeared in 1998 [6]. PMQR has been increasingly reported among different clinical strains in most parts of the world in the past decade. Generally, the genes responsible for PMQR have three mechanisms: (1) Quinolone resistance (*qnr*) genes encode proteins that block quinolones by target modification. Seven *qnr* families have been found in clinical isolates (*qnrA, qnrB, qnrS, qnrC, qnrD, qnrE*, and *qnrVC*) [7]. (2) The aminoglycoside acetyltransferase (*aac(6')-Ib-cr*) gene encodes an enzyme that modifies fluoroquinolones and aminoglycosides through acetylation and reduces drug activity [8]. (3) Efflux pump genes: quinolone efflux plasmid (*qepA*) and olaquindox (*OqxAB*), which excrete hydrophobic fluoroquinolones [9]. The resistance pattern to fluoroquinolones has changed among all *Enterobacteriaceae* and *P. aeruginosa* due to the dissemination of PMQR genes. Chromosomal-mediated resistance is only transmitted vertically from generation to generation, while PMQR genes can be transmitted horizontally as well as vertically, resulting in the rapid global dissemination of these genes [7].

Extended-spectrum β-lactamases (ESBLs) are one of the common mechanisms of resistance in *P. aeruginosa*. They are also carried on plasmids and can be easily transmitted among different bacteria. ESBLs are serine B-lactamases of class A, one of the four major classes of the Ambler classification of B-lactamases [10]. *The blaTEM, blaCTX-M*, and *blaSHV* genes are the most prevalent enzymes in this class A and have been reported in *P. aeruginosa* strains [11]. Despite the increased importance of quinolone resistance and its plasmid-mediated mechanism in the last decade, as well as several reports from Egypt detecting an increased incidence of PMQR genes among *Escherichia coli (E. coli)* [12] and *Klebsiella pneumoniae* [13], few studies have addressed this issue with *P. aeruginosa* in Egypt. The emergence of bacterial strains carrying both ESBL and PMQR genes is now a serious global health threat [14]. Due to the increased worldwide occurrence and diversity of PMQR and ESBL genes among *P. aeruginosa* strains, it is now necessary to identify these genes for the proper initiation of antibiotic therapeutic regimens. To the best of our knowledge, there is a dearth of data regarding the co-existence of PMQR and ESBL genes among *P. aeruginosa* in Egypt. In this study, we aimed to determine antimicrobial resistance patterns, particularly to fluoroquinolones and cephalosporins, among *P. aeruginosa* isolates obtained from different clinical specimens from patients admitted to Minia University Hospitals, Egypt. Then molecular screening of resistant isolates for PMQR and ESBL genes was carried out to study the co-existence of different resistance determinants.

## Patients and methods

### Study design

This study was carried out in the Department of Medical Microbiology and Immunology, Faculty of Medicine, Minia University, Egypt. A total of 56 *P. aeruginosa* isolates were obtained from 330 different clinical specimens of patients admitted to Minia University Hospitals in the period from January 2023 to May 2023. Patients with any history of antibiotic use two weeks before specimen collection were excluded. The study was performed in accordance with the guidelines of the Declaration of Helsinki. Informed consent was obtained from all patients. The study protocol was approved by the Ethics Committee of the Faculty of Medicine of Minia University (Approval No. 631/2023).

### Bacterial isolation and identification

A total of 56 non-repetitive *P. aeruginosa* isolates were recovered from different clinical specimens as follows: urine (*n* = 31), wound swabs (*n* = 18), ear swabs (*n* = 5), and eye swabs (*n* = 2). Urine samples were collected under complete aseptic precautions in sterile containers, and other clinical specimens were collected aseptically in sterile swabs with transport media. They were transported within 2 h to the microbiology laboratory for immediate examination. Samples were streaked on MacConkey’s agar and nutrient agar (Oxoid, UK) and were then purified on cetrimide agar (Scharlu, Spain). After overnight incubation at 37 °C, all isolated colonies were identified morphologically (Gram stain) and by standard biochemical tests (sugar fermentation, catalase +, oxidase +, indole -, methyl red -, citrate +, H_2_S production -, oxidative, and motility) [15]. All isolates were identified and confirmed as *P. aeruginosa*. Then the identified isolates were inoculated in Trypticase soy broth (Oxoid, UK), mixed with sterilized glycerol (20%) after 24 h of incubation, and stored at -20 °C for further testing.

### Antibiotic susceptibility testing

The Kirby-Bauer disc diffusion technique was used to identify antimicrobial resistance among *P. aeruginosa* isolates using Muller-Hinton agar (Oxoid, England) according to Clinical Laboratory Standard Institute (CLSI) guidelines [16]. The following commercially available discs (Oxoid, England) were used, including: [ciprofloxacin (CIP, 5 µg), representing the second-generation fluroquinolone; levofloxacin (LEV, 5 µg), representing the third-generation fluroquinolone]; aminoglycosides [amikacin (AMK, 30 µg)], cephalosporins [ceftriaxone (CRO, 30 µg) and cefepime (CEF, 30 µg)]; and carbapenem [imipenem (IMP, 10 µg)]. *P. aeruginosa* ATCC27853 was used as a quality control strain.

### DNA extraction

The DNA of 56 *Pseudomonas* isolates was extracted using a modified boiling technique by the heat shock method [17]. After centrifugation of 1.5 ml of overnight bacterial culture at 13,000 rpm for 5 min at 4 °C, the supernatant was removed carefully, and the pellet was suspended in 200 µl of sterile distilled water. The suspended pellet was then put for 15 min in a water bath and immediately shocked by ice-cooling for 10 min. Lastly, centrifugation at 13,000 rpm for 5 min at 4 °C was done, and the supernatant containing genomic DNA was transferred into a new tube for subsequent PCR amplification. The extracted DNA was used immediately or stored at -20˚C until used.

### Detection of PMQR genes among the quinolone-resistant isolates

*Pseudomonas* isolates, phenotypically resistant to any of the tested quinolones, were screened for the following six genes: quinolone resistance genes (*qnrA, qnrB, qnrC*, and *qnrS*), the quinolone efflux gene (*qepA*), and the quinolone modifying enzyme gene (*acc(6')-Ib-cr*). Single PCR reactions were used for the amplification of each of the PMQR genes. Each PCR reaction was performed in a total volume of 25µL containing 12.5µL of Hot Start Green PCR Master Mix (Applied Biosystems™, USA), 1µL (10 pmol) of each primer, 3µL (300 ng/mL) of DNA template, and 7.5µL of nuclease-free water. The PCR conditions used for amplification of *qnr* genes (qnrA, qnrB, and *qepA*) were: Initial denaturation at 95 °C for 15 min followed by 30 cycles of denaturation at 95 °C for 1 min, annealing at 55 °C for 1 min, and 72 °C for 5 min, and one cycle of final elongation at 72 °C for 5 min. The thermocycling conditions for the other genes (*qnrS, qnrC*, and *acc(6')-Ib-cr*) were the same with changed annealing temperatures. The used primer sequences, annealing temperatures, and sizes of amplified fragments of the studied genes [18–22] are listed in Table 1. The PCR products were resolved on 1.5% Agarose gel containing ethidium bromide dye, and the gel was visualized under a UV transilluminator.

**Table 1:** PCR primers used in the study

Gene	Primers (5’ to 3’)	Amplicon size (bp)	Annealing Temperature	Reference
*qnrA*	F- AGAGGATTTCTCACGCCAGG R- TGCCAGGCACAGATCTTGAC	580	55 °C	17
*qnrB*	F- GGMATHGAAATTCGCCACTG R- TTTGCYGYYCGCCAGTCGAA	264	55 °C	17
*qnrS*	F: GCAAGTTCATTGAACAGGGT R: TCTAAACCGTCGAGTTCGGCG	428	66.7 °C	17, 19
*qnrC*	F: GGGTTGTACATTTATTGAATC R: TCCACTTTACGAGGTTCT	447	50 °C	20
*qepA*	F: ACATCTACGGCTTCTTCGTCG R: AACTGCTTGAGCCCGTAGATC	502	55 °C	18
*acc(6')-Ib* *-cr*	F: TTGCGATGCTCTATGAGTGGCTA R: CTCGAATGCCTGGCGTGTTT	482	63 °C	21
*blaTEM*	F-ATGAGTATTCAACATTTCCG R-CCAATGCTTAATCAGTGAGG	858	50 °C	22
*blaSHV*	F-CGCCGGGTTATTCTTATTTGTCGC R-TCTTTCCGATGCCGCCGCCAGTCA	1017	55 °C	23
*universal blaCTX-m*	F-SCSATGTGCAGYACCAGTAA R-CCGCRATATGRTTGGTGGTG	554	61 °C	24

Abbreviations: bp (base pair)

### Detection of ESBL genes

All *P. aeruginosa* isolates that were resistant to cephalosporins (50 isolates) were tested by the PCR assay to determine the β-lactamase-encoding genes (*blaTEM, blaSHV*, and *blaCTX-M*) using the specific primers [23–25]. PCR amplification was performed in a thermocycler (Biometra, UNO II, Goettingen, Germany) as follows: 94 °C for 4 min; 35 cycles of 1 min at 94 °C, 1 min at a specific temperature for each primer, and 1 min at 72 °C; and a final extension step of 10 min at 72 °C. The primer sequences and annealing temperatures used are described in Table 1. PCR products were electrophoresed on a 1.5% agarose gel at 100 V, stained with ethidium bromide dye, and finally visualized with a UV transilluminator system. Positive resistant strains from a previous study in our laboratory were obtained and served as the positive control [26].

### Statistical analysis

Statistical analysis of clinical and laboratory data of the studied subjects was performed by IBM SPSS software (version 19.0). A *P* value of < 0.05 was considered as an indication of statistical significance.

## Results

### Demographic characteristics of the study subjects

A total of 56 *P. aeruginosa* isolates were obtained from 330 (16.9%) patients with different clinical infections. In our study, *P. aeruginosa* was mostly isolated from patients with urinary tract infection (UTI) infection (31/210, 14.7%), wound infection (18/85, 21.2%), otitis media (5/20, 25%), and eye infection (2/15, 13.3%). *P. aeruginosa* infections were more common in males (36/56, 64.3%) than females (20/56, 35.7%), and their ages ranged from 18 to 66 years.

### Antimicrobial susceptibility of P. Aeruginosa isolates

From all 56 *P. aeruginosa* clinical isolates tested for antimicrobial susceptibility, the most predominant resistance was against third-generation cephalosporins: ceftriaxone (50/56, 89.3%) and cefepime (32/56, 57.1%). Amikacin showed moderate resistance (19/56, 33.9%). The highest susceptibility rate was found for imipenem (44/56, 78.6.1%).

Regarding fluoroquinolone susceptibility, a total of 22/56 (39.3%) of studied *P. aeruginosa* isolates were resistant to one or both tested fluoroquinolones, with 20/56 (35.7%) resistance to ciprofloxacin, 17/56 (30.3%) resistance to levofloxacin, and 12/56 (21.4%) resistance to both ciprofloxacin and levofloxacin. The phenotypic fluoroquinolone-resistant *P. aeruginosa* was recovered from urine (11/22, 50%), wounds (8/22, 36.4%), and ear discharge (3/22, 13.6%). There were significant differences between the quinolone-resistant and quinolone-susceptible isolates in terms of the antibiotic resistance rates of amikacin, imipenem, and cefepime (*P* value < 0.0001). Table 2.

**Table 2:** Distribution of antibiotic resistance patterns and ESBL genes between phenotypic fluoroquinolone resistance and sensitive *P. aeruginosa* isolates

Variable		Fluoroquinolone resistant isolates Total (22)	Fluoroquinolone sensitive isolates Total (34)	*P* value
Cefepime	Resistant	1986.4%	1338.2%	< 0.0001*
	Sensitive	313.6%	2161.8%	
Ceftriaxone	Resistant	2195.5%	2985.3%	0.230*
	Sensitive	14.5%	514.7%	
Amikacin	Resistant	1986.4%	0.0%	< 0.0001*
	Sensitive	313.6%	34100.0%	
Imipenem	Resistant	1254.5%	0.0%	< 0.0001*
	Sensitive	1045.5%	34100.0%	
*blaTEM*	Positive	731.8%	514.7%	0.127
	Negative	1568.2%	2985.3%	
*blaCTX-M*	Positive	1359.1%	720.6%	0.003*
	Negative	940.9%	2779.4%	

* (significant *P* value)

### Detection of PMQR genes

**Fig. 1:**
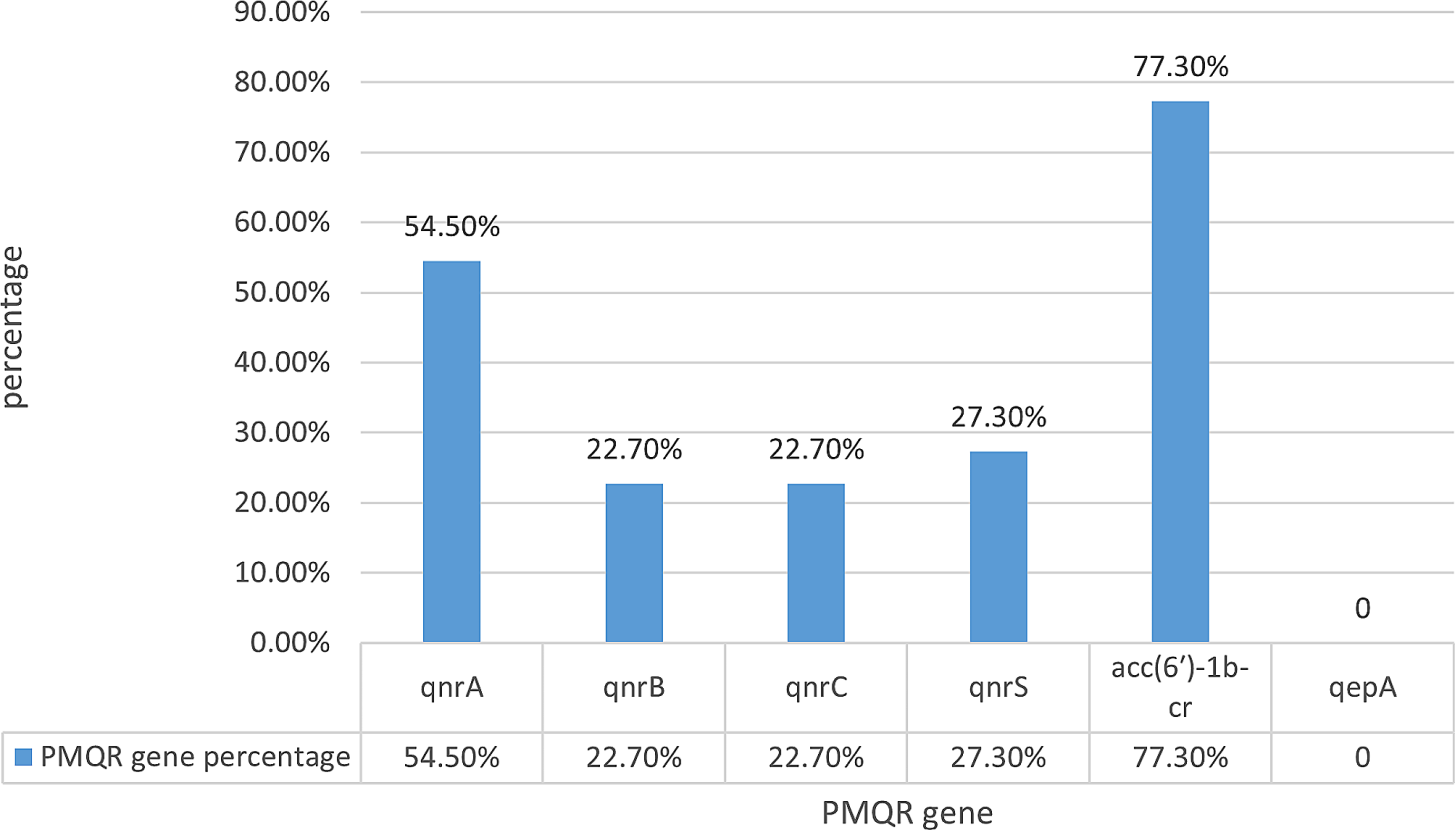
Frequencies of PMQR genes among fluoroquinolones-resistant *P. aeruginosa* isolates [The *acc(6')-Ib-cr* gene was the most prevalent PMQR gene among isolates (17/22, 77.3%); Among the studied *qnr* genes, the *qnrA* gene was the most predominant, at 54.5% (*n* = 12), followed by *qnrS* gene at 27.3% (*n* = 6), and then each of *qnrB* and *qnrC* at 22.7% (*n* = 5); the *qepA* gene was not detected in the studied isolates]

The frequencies of the studied six PMQR genes among all phenotypic fluoroquinolone-resistant *P. aeruginosa* isolates are shown in Fig. 1. Out of 22 fluoroquinolone-resistant *P. aeruginosa* isolates, 20 (90.9%) were positive for one or more of the PMQR genes. The results showed that *acc(6')-Ib-cr* was the most prevalent PMQR gene among isolates (17/22, 77.3%). The *qnr* genes occurred in 72.7% (*n* = 16/22) of isolates. The *qnrA* gene was the most predominant, at 54.5% (*n* = 12), followed by *qnrS* gene at 27.3% (*n* = 6), and then each of *qnrB* and *qnrC* at 22.7% (*n* = 5). Association of *acc(6')-Ib-cr* with *qnr* genes was detected in 65% (13/20) of positive PMQR isolates, and 9 isolates (45%) showed coexistence of more than one *qnr* gene with *acc(6')-Ib-cr*. The efflux pump gene *qepA* was not detected in the studied isolates. The distribution of PMQR genes among different specimen sources of *P. aeruginosa* isolates is illustrated in Table 3, with a significantly higher rate of the *qnrS* gene among isolates from ear discharge than urine and wounds (*P* value = 0.009).

In the current study, *P. aeruginosa* isolates positive for PMQR genes exhibited 11 different gene profiles. Table 1 Supplementary More than one gene was detected in 14/20 (70%) of positive isolates. It was found that one isolate (5%) harbored 4 genes, 9 isolates (45%) harbored 3 genes, and 4 (20%) carried 2 genes. The most frequently detected combined gene profiles carried 3 genes: gene profile (*qnrA + qnrS + acc(6')-Ib-cr*) and gene profile (*qnrA + qnrC + acc(6')-Ib-cr*). The *acc(6')-Ib-cr* gene was the most prevalent single gene profile, while *qnrS* and *qnrC* genes were not detected alone in any isolate. All *qnrS* gene-positive isolates carried *acc(6')-Ib-cr*. Gene profiles carrying more than two genes were prevalent in *P. aeruginosa* isolates from wounds and ear swabs.

**Table 3:** Distribution of PMQR genes among different specimens of *P. aeruginosa* isolates

PMQR gene	Type of Specimen						*P* value
	Urine (11 isolates)		Wound 8 isolates		Otitis media 3 isolates		
	Count	%	Count	%	Count	%	
*qnrA*	4	36.4%	5	62.5%	3	100.0%	0.124
*qnrB*	4	36.4%	0	0.0%	1	33.3%	0.156
*qnrC*	2	18.2%	3	37.5%	0	0.0%	0.367
*qnrS*	2	18.2%	1	12.5%	3	100.0%	0.009*
*acc(6')-Ib-cr*	7	63.6%	7	87.5%	3	100.0%	0.283

Abbreviations: PMQR (Plasmid-mediated quinolone resistance), * (significant *P* value)

### Detection of ESBL genes

**Fig. 2:**
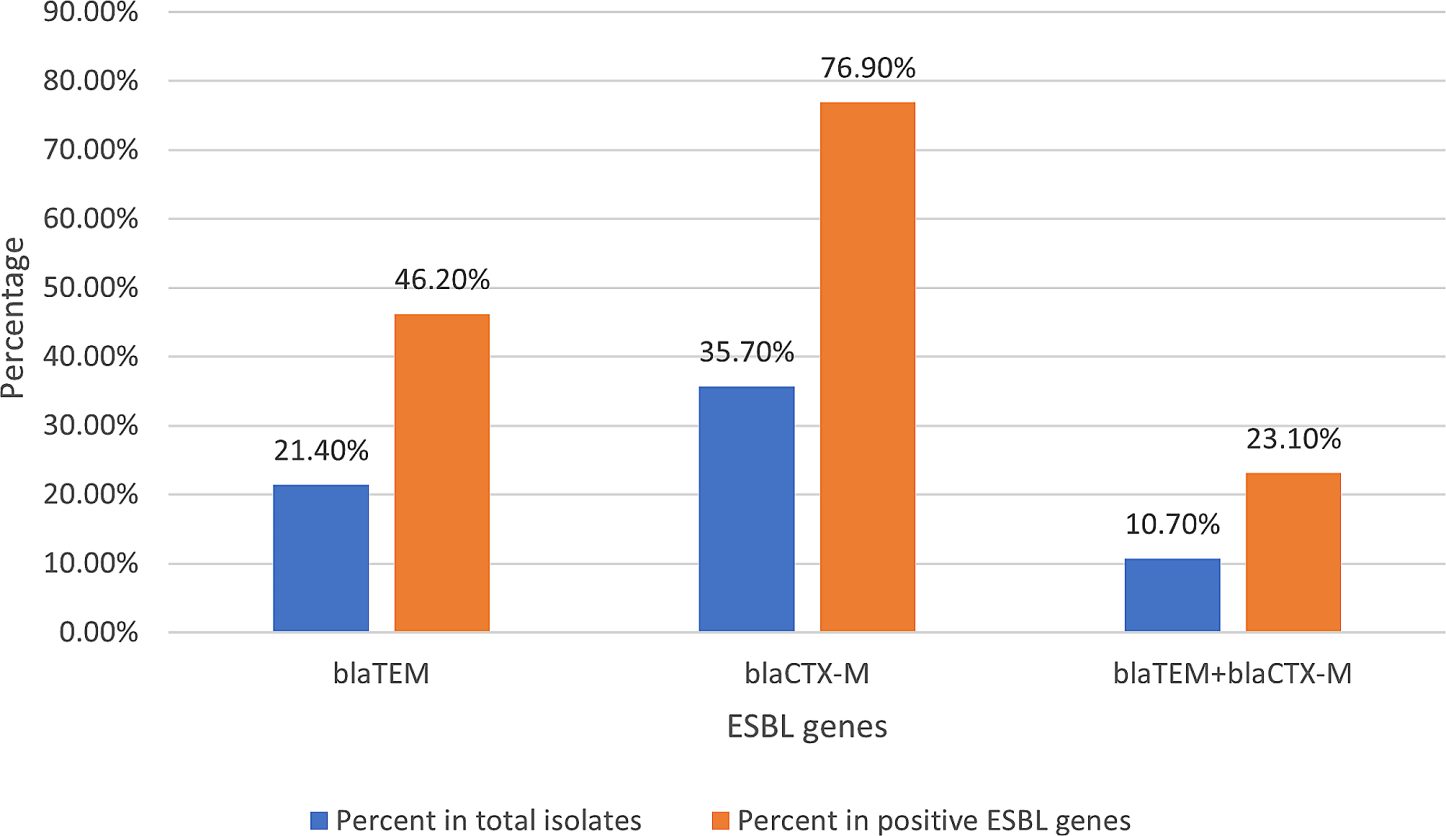
Frequencies of ESBL genes among studied *P. aeruginosa* isolates [The *blaCTX-M* was the most frequent ESBL gene (20/26, 76.9% of ESBL gene-positive isolates;20/56. 35.7% of all studied isolates), followed by *blaTEM* (12/26, 46.2% of ESBL gene-positive isolates; 12/56 21.4% of all studied isolates). Co-carriage of *blaTEM* and *blaCTX-M* was found in 6 isolates (23.1% of ESBL gene-positive isolates and 10.7% of all studied isolates)]

Among 50 *P. aeruginosa* isolates initially described as resistant to cephalosporins (ceftriaxone and/or cefepime), ESBL genes were detected in 26 isolates (52%). The frequency of ESBL genes among the isolates showed that *blaCTX-M* (20/26, 76.9% of ESBL gene-positive isolates) was the most frequent ESBL gene, followed by *blaTEM* (12/26, 46.2%). No isolates carried *blaSHV*. Co-carriage of *blaTEM* and *blaCTX-M* was found in 6 isolates (23%). Figure 2 The distribution of ESBL genes among different specimen sources of *P. aeruginosa* isolates was as follows: 12 from urine (46.2%), 8 from wounds (30.8%), 5 from ear discharge (19.2%), and 1 from eye discharge (3.8%). Positive isolates for ESBL genes showed significantly higher resistance to tested fluoroquinolones, aminoglycosides, and carbapenems than genotypic ESBL-negative isolates. Table 4 The rates of ciprofloxacin and levofloxacin resistance in ESBL-producing isolates were 15/26 (57.7%) and 13/26 (50%), respectively. Fluroquinolone-resistant isolates demonstrated significantly higher carriage of the *blaCTX-M* gene than sensitive isolates (*P* value = 0.003). Table 2.

**Table 4:** Distribution of antibiotic resistance patterns and PMQR genes between ESBL gene positive and negative isolates among cephalosporin-resistant *P. aeruginosa* isolates

Variable		ESBL genes positive isolates (26)	ESBL genes negative isolates (24)	*P* value
Ciprofloxacin	Resistant	15 (57.7%)	4(16.7%)	0.003*
	Sensitive	11 (42.3%)	20 (83.3%)	
Levofloxacin	Resistant	13(50%)	4(16.7%)	0.013*
	Sensitive	13 (50%)	20 (86.7%)	
Amikacin	Resistant	15 (57.7%)	4 (13.3%)	0.003*
	Sensitive	11(42.3%)	20(86.7%)	
Imipenem	Resistant	11(42.3%)	1 (4.2%)	0.002*
	Sensitive	15(57.7%)	23 (95.8%)	
*qnrA*	Positive	11 (42.3%)	1(4.2%)	0.002*
	Negative	15(57.7%)	23(95.8%)	
*qnrB*	Positive	4(15.4%)	1(4.2%)	0.187
	Negative	22(84.6%)	23 (95.8%)	
*qnrC*	Positive	5 (19.2%)	0 (0%)	0.024*
	Negative	21 (80.8%)	24(100%)	
*qnrS*	Positive	5 (19.2%)	1(4.2%)	0.101
	Negative	21(80.8%)	23(95.8%)	
*acc(6')-Ib-cr*	Positive	13(50%)	3(12.5%)	0.005*
	Negative	13 (50%)	21(87.5%)	

Abbreviations: ESBL (Extended Spectrum Beta-Lactamases), ^*^P value is significant

### Coexistence of PMQR and ESBL genes

In this study, at least one ESBL gene was found in 15 isolates (75% of PMQR-positive isolates). We found that PMQR genes could co-exist with *blaCTX-M* and *blaTEM*. (Table 5). The *acc(6')-Ib-cr* gene showed the highest association with ESBL genes (13 isolates), followed by the *qnrA* gene (11 isolates). The positive ESBL gene isolates showed significantly higher carriage of *qnrA*, *acc(6')-Ib-cr*, *and qnrC* (*P* values were 0.002, 0.005, and 0.024, respectively). Table 4 Most isolates that co-carried PMQR and ESBL genes were isolated from urine (7 isolates, 46.7%), followed by wounds (5 isolates, 33.3%), and ear discharge (3 isolates, 20%). The correlation matrix of the detected PMQR and ESBL genes indicated overall positive correlations. The strongest and most highly significant correlation was between *qnrA* and *acc(6')-Ib-cr* (*r* = 0.602) and between *qnrA* and *blaCTX-M* (*r* = 0.519). Table 6.

**Table 5:** Co-existence of ESBLs and PMQR genes in *P. aeruginosa* isolates

No. of genes	PMQR gene					ESBL gene		Source of isolate	No. of isolates
	*qnrA*	*qnrB*	*qnrC*	*qnrS*	*acc(6')-Ib-cr*	blaTEM	blaCTX-M		15
2 genes	+	-	-	-	-	-	+	U	1
	-	-	-	-	+	-	+	W	1
3 genes	+	-	-	-	+	-	+	W	1
	+	-	-	-	+	+	-	U	1
	-	-	-	+	+	-	+	U	1
4 genes	+	-	-	-	+	+	+	U	1
	+	-	+	-	+	-	+	W	1
	+	-	-	+	+	-	+	Ear	1
	-	+	+	-	+	+	-	U	1
	+	+	+	-	-	-	+	U	1
5 genes	+	-	+	-	+	+	+	W	2
	+	+	-	+	+	-	+	Ear	1
	-	+	-	+	+	+	+	U	1
	+	-	-	+	+	+	+	Ear	1

Abbreviations: PMQR (Plasmid mediated quinolone resistance), ESBL (Extended Spectrum Beta-Lactamases), U(urine), W(wound)

**Table 6:** Correlation matrix (r2) between the different PMQR and ESBL genotypes

Variable	qnrA	qnrB	qnrC	QnrS	acc(6')-Ib-cr	blaTEM	blaCTX-M
*qnrA*	1	0.142	0.447**	0.382**	0.602**	0.258	0.519**
*qnrB*		1	0.341*	0.296*	0.202	0.142	0.159
*qnrC*			1	-0.108	0.338*	0.294*	0.289*
*qnrS*				1	0.525**	0.101	0.344**
*acc(6')-Ib-cr*					1	0.318*	0.399**
*blaTEM*						1	0.156
*blaCTX-M*							1

* Correlation is significant at the 0.05 level (2-tailed). ** Correlation is significant at the 0.01 level (2-tailed).

## Discussion

The increasing local reports of PMQR genes and their association with ESBLs among *Enterobacteriaceae* are alarming and can be a major factor in the increasing prevalence of these genes among *Pseudomonas* strains [12, 27]. Extensively improper use of broad-spectrum antibiotics in our country has exacerbated this problem. In the current study, *P. aeruginosa* isolates were detected in 19.3% of the collected clinical specimens. That was similar to the results reported by Saki et al. [28] and fewer than Farhan et al. [29]. Regarding the specimen site, *P. aeruginosa* was isolated from 31 of 210 (14.3%) urine samples, 18 of 85 (21.2%) wound swabs, five of 20 (25%) ear swabs, and two of 15 (13.3%) eye swabs. Farhan and his colleagues [29] found a similar prevalence among wound swabs (19.5%) and a higher prevalence in urine (20%) and ear swabs (68.4%).

Twenty-two of the isolates (39.3%) were resistant to at least one or both tested fluoroquinolones (ciprofloxacin and levofloxacin). This result was like previous local reports [30–32] and worldwide results from Poland [33] (39.6%), Brazil [34] (42%), Saudi Arabia [20] (42.4%), Nigeria [35] (35.5%), and China [36] (32%). In contrast to our findings, Saki et al. [28] and Molapour et al. [37] from Iran reported higher resistance of clinical *P. aeruginosa* isolates to fluoroquinolones. However, Abbas and his colleagues [38] found high susceptibility (> 90%) of *P. aeruginosa* isolated from urinary tract infections to ciprofloxacin and levofloxacin. This variation in resistance patterns among isolates could be due to differences in geographical regions, environmental conditions, and antibiotic prescription patterns among different countries. Also, variation in resistance rates in the same country may be attributed to the different infection types and the disease severity among patients, affecting the drug susceptibility of *P. aeruginosa* isolates. Fluoroquinolones are one of the most common empiric therapeutic regimens for UTI in our country, explaining that more than 50% of *P. aeruginosa* isolates resistant to fluoroquinolones were from urine.

In the current study, 90.9% of fluoroquinolone-resistant *P. aeruginosa* isolates were positive for one or more of the PMQR genes. The *acc(6')-Ib-cr* gene was the most prevalent PMQR gene among isolates (77.3%). The *qnr* genes were detected in 72.7% of isolates, with the *qnrA* gene being the most predominant (54.5%), followed by the *qnrS* gene (27.3%), then *qnrB* and *qnrC* (each 22.7%). In agreement with this result, El-Badawy and his colleagues [20] in Saudi Arabia detected PMQR genes in 37 of 39 isolates (94%), but *qnrS* was the most prevalent (79.5%). Rajaei et al. [39] from Iran agreed with our result that the *qnrA* gene was the most frequent among *qnr*-positive isolates. Regarding data about PMQR among *P. aeruginosa* in Egypt, Abdelmegeed and his colleagues (2017) [40] detected a similar percent of PMQR genes (89%) in *Pseudomonas* isolates with different frequencies (*qnrS* 41%, *qnrB* 22%, *qnrA* 9%, and *acc(6')-Ib-cr* 22%). On the other hand, Ali et al. (2018) [41] reported PMQR genes in 42.9% of quinolone-resistant *P. aeruginosa*, with a predominance of *acc(6')-Ib-cr* and a low percent of *qnr* genes *(qnrS and qnrA)*. In an earlier study by Saleh et al. [30], *qnr* genes (*qnrS* and *qnrB)* were encountered in only 4.5% of isolates. In a recent study from Iran, the qnr genes were detected in 37.5% of quinolone-resistant *P. aeruginosa* isolates, with frequency rates as follows: *qnrB* (29.2%), *qnrA* (25.8%), and *qnrS* (20.8%) [28]. However, Venkataramana and his colleagues [42] from India reported that 68.5% of clinical strains of *P. aeruginosa* harbored PMQR genes. The results of the current study disagree with earlier studies from Iran [37] and Turkey [43] that did not detect any *qnr* gene in quinolone-resistant *P. aeruginosa*. The higher prevalence of PMQR genes in our study may indicate their increasing rate and reflect that there is extensive and inappropriate use of fluoroquinolones, resulting in the emergence of more resistant isolates in our hospital settings.

The prevalence of the *aac(6’)-Ib-cr* gene was 77.3%; this result agreed with similar studies conducted in India (77.6%) [42] and KSA (71.8%) [20]. High rates of this gene were also detected in several studies among *Enterobacteriaceae* [12, 13] and *P. aeruginosa* in Egypt [41]. The high prevalence of this gene could be attributed to the excessive use of aminoglycosides or hydrophilic fluoroquinolones in our country. The *aac (6’)-Ib-cr* gene plays a major potential role in the emergence of clinical ciprofloxacin resistance.

The efflux pump gene *qepA* was not detected in any of the studied isolates, as reported in many previous studies in Egypt [30], Saudi Arabia [20], Iran [28], India [42], and Turkey [43]. Two isolates of quinolone-resistant *P. aeruginosa* did not carry any tested PMQR genes. The quinolone resistance of these isolates can be due to chromosomal resistance or another PMQR gene not investigated in our study.

The remarkable result of the current study was the high co-occurrence of PMQR genes among the quinolone-resistant isolates. Association of *acc(6')-Ib-cr* with *qnr* genes was detected in 65% of positive PMQR isolates. All *qnrS* gene-positive isolates carried *acc(6')-Ib-cr*. Co-carriage of more than one *qnr* gene with *acc(6')-Ib-cr* was also found in 45% of isolates carrying PMQR. In accordance with these results, the association of PMQR genes among *P. aeruginosa* isolates has been reported in several studies from various countries, including KSA [20], India [42], and China [44]. Recently, the coexistence of three PMQR genes (*aac(6')-Ib-cr, qnrS2*, and *oqxAB*) was also reported on a multiple resistance plasmid in *E. coli* in China [45]. This finding is an alarm for the need for the implementation of strict antibiotic policies and infection control strategies to limit the spread of these emerging strains. Different studies associate the presence of the *qnr* genes with long hospital stays and increased 30-day mortality [46]. In support of this, the poorest clinical response and fatal outcomes in murine models of urinary tract infection and pneumonia due to the presence of PMQR genes have also been reported [47].

The co-carriage of PMQR genes has changed the fluoroquinolone resistance pattern of *Enterobacteriaceae* and *P. aeruginosa*. Although the long-established view that PMQR genes produce only low-level resistance to fluoroquinolones which does not exceed the clinical susceptibility level, they can complement the chromosomal resistance mechanism to reach clinical resistance through the selection of higher-level resistance strains [7]. The enzyme encoded by the *aac(6')-Ib-cr* gene facilitates the selection of high ciprofloxacin-resistant chromosomal mutants and converts the low-level resistance of fluoroquinolone mediated by this enzyme to high-level resistance when coexisting with qnr proteins. More importantly, there is evidence for emerging clinical quinolone resistance without topoisomerase mutations in a resistant *E. coli* strain carrying PMQR genes (*qnrS1* and *oqxAB*, as well as overexpressing other efflux pump genes) [48]. Also, reports of PMQR genes, either alone or in combination, mediate ciprofloxacin resistance in *Salmonella* isolates in the absence of any chromosomal mutations [49]. PMQR genes may also result in alterations in the expression levels of specific genes controlling the susceptibility levels of unrelated agents [46].

In the current study, we examined the prevalence of ESBL genes among isolates resistant to cephalosporin. Detection of ESBL production among *P. aeruginosa* by molecular techniques is more accurate, as *AmpC* production by isolates may interfere with or hide the detection of ESBLs by phenotypic tests [50]. We found that 46.4% of all *P. aeruginosa* isolates and 52% of cephalosporin-resistant *P. aeruginosa* isolates carry ESBL genes. *The blaCTX-M* gene was the most frequent ESBL (76.9%), followed by *blaTEM* (46.2%). No isolates carried *blaSHV*. Co-carriage of *blaTEM* and *blaCTX-M* was found in 23% of isolates carrying ESBL genes. In line with our results, Goudarzi et al. from Iran [51], Nasser et al. from Yemen [52], and Ejaz from Pakistan [53] reported that *blaCTX-M* was the more prevalent ESBL gene than TEM and SHV types among *P. aeruginosa*. In contrast to our results, *blaTEM* was the most detected in West Iran among *P. aeruginosa* isolates from coronavirus disease-19 patients [54]. In an earlier study in Minia, Egypt, Noha et al. (2015) detected *blaTEM* only in 12.5%, and in agreement with our results, no isolate had *the blaSHV* gene [55]. However, Farhan and his colleagues in 2019 found *CTX-M15* among 55.5% of ESBL-producing *P. aeruginosa* [29]. Before one decade, *blaTEM* and *blaSHV* were the predominant types of ESBLs, and *blaCTX* was scarcely detected in a random pattern. Today, alarming dissemination of *CTX-M*-type enzymes was found to be mainly associated with the emerging *E. coli* ST131 clone that usually harbors IncF plasmids [10]. A high prevalence of this clone was recently reported in Egypt among isolates from UTIs or colonizing intestines [56]. This may explain the increasing prevalence of the *blaCTX-M* gene in recent studies when compared with earlier ones.

PMQR genes have been found on heterogenic plasmids varying in size and incompatibility specificity, explaining the dissemination of this resistance around the world due to the spread of multiple plasmids between different bacterial hosts and multiple independent acquisitions of these plasmids [57]. The *aac(6')-Ib-cr* variant can be carried on plasmids of different incompatibility groups, but most commonly IncF type. IncF plasmids carry a wide range of resistance genes to major classes of antimicrobials. ESBL genes, especially those of the CTX-M type, PMQR genes, and genes encoding aminoglycoside-modifying enzymes *(acc(6')-Ib-cr)*, are the major trait of genes carried by IncF plasmids [58]. These plasmids are now considered a pandemic due to their widespread detection in different countries among different bacteria from different sources and origins. IncF plasmid carrying the *aac(6')-Ib-cr* gene has been reported in *P. aeruginosa* [59]. From this data, IncF plasmids play a major role in the global dissemination of different resistance genes. Megaplasmids that can encode a range of antimicrobial resistance and virulence genes are now of emerging interest in nosocomial infections associated with *P. aeruginosa* [60].

The co-existence of PMQR genes with *blaCTX-M* and *blaTEM* was found in our study (Table 5). About 75% of PMQR-positive isolates harbored at least one ESBL gene. The *acc(6')-Ib-cr* gene showed the highest association with ESBL genes (13 isolates), followed by the *qnrA* gene (11 isolates). Co-carriage of the *acc(6')-Ib-cr* gene with one or more of the *qnr* and ESBL genes was highly prevalent, representing half of positive ESBL gene isolates. Most isolates that co-carried PMQR and ESBL genes were isolated from urine (46.7%), followed by wounds and ear discharges. The genes coding for both PMQR and ESBL are usually carried on the same plasmid, with higher chances of transmission among different members of the *Enterobacteriaceae* family and *P. aeruginosa* strains. The widespread co-existence of these genes in our study supports the possibility of the presence of circulating plasmids carrying those resistance genes. *P. aeruginosa* is capable of rapidly developing resistance to antibiotics through molecular evolution. Excessive and inappropriate use of antibiotics in both medicine and animal feedstuffs produced selective pressure, resulting in an increased rate of plasmid-mediated horizontal gene transfer and the spread of antibiotic resistance genes. There is evidence that the subinhibitory concentrations of antibiotics and quorum-sensing inhibitory antibiotics resulted in a significant increase in the conjugative transfer of resistance genes from *E. coli* to *pseudomonas* [61]. The co-existence of ESBLs and PMQR genes increased multidrug-resistant *P. aeruginosa* strains, causing serious infections because of restrictions on the antibacterial agent’s choice, therapeutic failures, high public health costs, increased morbidity, and mortality.

The limitation of our study was the low sample size. From the results of the antibiogram, we found that carbapenems followed by aminoglycosides showed higher susceptible results than fluoroquinolones and cephalosporins. The antibiotic regimens used to treat *P. aeruginosa* infections need to be updated.

## Conclusions

Our study detected a high prevalence of PMQR genes (90.9%) among phenotypic fluoroquinolone-resistant *P. aeruginosa* isolates. The *acc(6')-Ib-cr* gene was the most prevalent PMQR gene and exhibited high co-carriage of multiple *qnr* genes among the studied isolates. The *blaCTX-M* gene was the predominant ESBL gene among isolates. The study showed the co-existence of PMQR and ESBL genes among clinical *P. aeruginosa* from different infections, with 75% of PMQR-positive isolates harboring at least one ESBL gene. The detected ESBL and PMQR genes showed generally positive correlations. The correlations between *qnrA* and *blaCTX-M* (*r* = 0.519) and *acc(6')-Ib-cr* (*r* = 0.602) were the greatest and most significant. This increases the risk of potential dissemination of MDR-resistant strains in hospital settings and emphasizes the need to establish strict policies of antibiotic use to limit the prompt spread of these isolates. To update treatment guidelines for serious infections caused by these developing resistant *P. aeruginosa* strains, more research is required for the ongoing monitoring of resistance patterns and molecular screening for these enzymes.

### Electronic supplementary material

Below is the link to the electronic supplementary material.

Supplementary Material 1

## Data Availability

All data generated or analyzed during this study are included in this article.

## Abbreviations

*aac(6')-Ib-cr*: aminoglycoside acetyltransferase gene
CIP: Ciprofloxacin
CLSI: Clinical Laboratory Standard Institute guidelines
ESBLs: Extended-spectrum beta-lactamases
ExPEC: Extra-intestinal pathogenic *E. coli*
IncF: Incompatibility F
MDR: Multiple drug-resistant
MIC: Minimum inhibitory concentrations
*P. aeruginosa*: *Pseudomonas aeruginosa*
PMQR: Plasmid-mediated quinolone resistance
*qepA*: Quinolone efflux plasmid gene
*qnr*: Quinolone resistance genes
UTI: Urinary tract infection
WHO: World Health Organization
