# Supplementary material for: Coexistence of plasmid-mediated quinolone resistance (PMQR) and extended-spectrum beta-lactamase (ESBL) genes among clinical Pseudomonas aeruginosa isolates in Egypt
Source: BMC Microbiol. 2024 May 21;24:175. doi: 10.1186/s12866-024-03319-z (PMC11106877; doi:10.1186/s12866-024-03319-z)
Supplement: Supplementary file 1 — Supplementary Material 1 [file 12866_2024_3319_MOESM1_ESM.docx]

Table 1 Different gene profiles of PMQR genes among phenotypic fluoroquinolone resistance *P. aeruginosa* isolates

| No of genes | Gene profile No. | Genes in profile | Source of isolate | | | No. of isolates |
| --- | --- | --- | --- | --- | --- | --- |
|  |  |  | Urine | Wound | Ear discharge |  |
| 1  gene | 1  2  3 | *acc(6ʹ)-Ib-cr*  *qnrA*  *qnrB* | 2  1  1 | 2  -  - | -  -  - | 4  1  1 |
|  |  |  |  |  |  | Total=6 |
| 2 genes | 4  5 | *qnrA+ acc(6ʹ)-Ib-cr*  *qnrS+ acc(6ʹ)-Ib-cr* | 2  1 | 1  - | -  - | 3  1 |
|  |  |  |  |  |  | Total=4 |
| 3 genes | 6  7  8  9  10 | *qnrA+ qnrS+ acc(6ʹ)-Ib-cr*  *qnrA+ qnrC+ acc(6ʹ)-Ib-cr*  *qnrB+ qnrS+ acc(6ʹ)-Ib-cr*  *qnrB+ qnrC+ acc(6ʹ)-Ib-cr*  *qnrA+ qnrB+ qnrC* | -  -  1  1  1 | 1  3  -  -  - | 2  -  -  -  - | 3  3  1  1  1 |
|  |  |  |  |  |  | Total=9 |
| 4 genes | 11 | *qnrA+ qnrB+ qnrS+ acc(6ʹ)-Ib-cr* | - | - | 1 | 1 |
| Total | | | 10 | 7 | 3 | 20 |

Table 2: Correlation matrix (r2) between the different PMQR and phenotypic fluoroquinolone resistance .

| Variable | Ciprofloxacin  resistance | Levofloxacin  resistance |
| --- | --- | --- |
| *qnrA* | 0.701** | 0.602** |
| *qnrB* | 0.420** | 0.338** |
| *qnrC* | 0.420** | 0.474** |
| *qnrS* | 0.456** | 0.399** |
| *acc(6ʹ)-Ib-cr* | 0.805** | 0.831** |

* Correlation is significant at the 0.05 level (2-tailed). ** Correlation is significant at the 0.01 level (2-tailed).


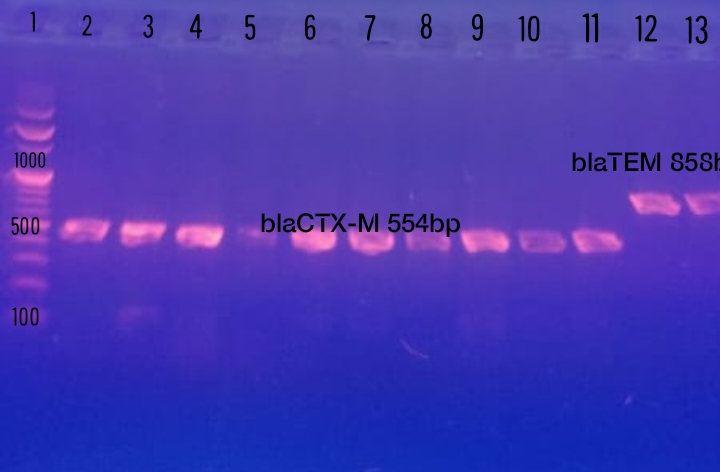


Figure 1 Screening of *blaTEM* and *blaCTX-M* genes in *P. aeruginosa* isolates

Lane 1, 100bp molecular marker (iNtRON Biotechnology, South Korea); Lane 2-11 show (*blaCTX-M* gene) 554bp; Lane 12, 13 (*blaTEM* gene) 858bp


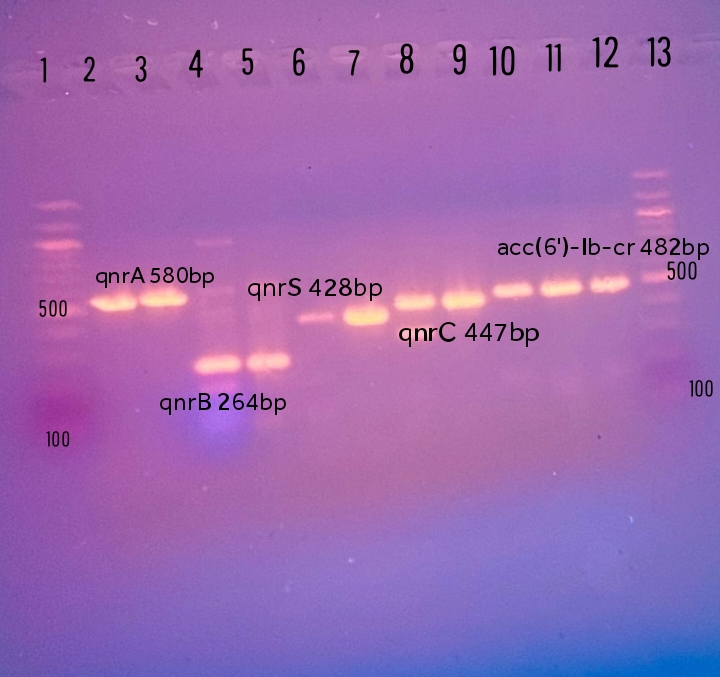


Figure 2 Screening of PMQR genes in *P. aeruginosa* isolates

Lane 1& 13, 100bp molecular marker (iNtRON Biotechnology, South Korea); Lane 2, 3 show (*qnrA* gene) 580 bp; Lane 4,5 (*qnrB* gene) 264bp; Lane 6, 7 (*qnrS* gene) 428bp; Lane 9 (*qnrC* gene) 447bp; Lane 10-12 (*acc(6’)Ib-cr* gene) 482bp
